# Overexpression of mitochondrial oxodicarboxylate carrier (ODC1) preserves oxidative phosphorylation in a yeast model of Barth syndrome

**DOI:** 10.1242/dmm.027540

**Published:** 2017-04-01

**Authors:** Maxence de Taffin de Tilques, Déborah Tribouillard-Tanvier, Emmanuel Tétaud, Eric Testet, Jean-Paul di Rago, Jean-Paul Lasserre

**Affiliations:** 1 Université de Bordeaux, Institut de Biochimie et Génétique Cellulaires, CNRS UMR 5095, 1 rue Camille Saint-Saëns, Bordeaux cedex 33077, France; 2 Université de Bordeaux, Laboratoire de biogenèse membranaire, CNRS UMR 5200, INRA Bordeaux Aquitaine BP81, 33883 Villenave d'Ornon Cédex, France

**Keywords:** Human mitochondrial disease, Oxidative phosphorylation, Barth syndrome, Oxodicarboxylic acid transport, Cardiolipin remodeling, Tafazzin

## Abstract

Cardiolipin (CL) is a diglycerol phospholipid mostly found in mitochondria where it optimizes numerous processes, including oxidative phosphorylation (OXPHOS). To function properly, CL needs to be unsaturated, which requires the acyltransferase tafazzin. Loss-of-function mutations in this protein are responsible for Barth syndrome (BTHS), presumably because of a diminished OXPHOS capacity. Here, we show that overexpressing Odc1p, a conserved oxodicarboxylic acid carrier located in the mitochondrial inner membrane, fully restores oxidative phosphorylation in a yeast model (*taz1Δ*) of BTHS. The rescuing activity involves the recovery of normal expression of key components that sustain oxidative phosphorylation, including cytochrome *c* and electron transport chain complexes IV and III, which are strongly downregulated in *taz1Δ* yeast. Interestingly, overexpression of Odc1p was also shown previously to rescue yeast models of mitochondrial diseases caused by defects in the assembly of ATP synthase and by mutations in the MPV17 protein that result in hepatocerebral mitochondrial DNA depletion syndrome. These findings define the transport of oxodicarboxylic acids across the inner membrane as a potential therapeutic target for a large spectrum of mitochondrial diseases, including BTHS.

## Abstract (precis)

**Summary:** Transport of oxodicarboxylic acid (ODC1) across the mitochondrial inner membrane is a potential target for the treatment of BTHS patients and other diseases caused by mitochondrial dysfunction.

## INTRODUCTION

Cardiolipin (CL) is an acidic diglycerophospholipid carrying two negative charges that is exclusively synthesized in mitochondria and mostly found in the mitochondrial inner membrane (IM) (Hostetler et al., 1972; Bligny and Douce, 1980; Hoch, 1992; Schlame and Haldar, 1993; Schlame et al., 2000; Joshi et al., 2009; Ikon et al., 2015). CL has a structure that is more flexible than other phospholipids because of two chiral carbons and four fatty acyl chains that are usually polyunsaturated. CL facilitates cristae formation (Xu et al., 2006; Acehan et al., 2011; Schlame et al., 2012) and establishes interactions with electron transport chain components (complexes I-IV), promoting their association into ‘supercomplexes’ or ‘respirasomes’, which is presumed to optimize respiration (Zhang et al., 2002; Pfeiffer et al., 2003; Bazan et al., 2013). CL also plays a role in many others processes including mitochondrial fusion (Joshi et al., 2012), fission (DeVay et al., 2009; Ban et al., 2010), protein import (Jiang et al., 2000; Gebert et al., 2009), iron-sulfur (Fe-S) biogenesis (Patil et al., 2013), mitophagy (Chu et al., 2013, 2014; Hsu et al., 2015; Li et al., 2015) and apoptosis (McMillin and Dowhan, 2002; Kim et al., 2004; Heit et al., 2011; Gonzalvez et al., 2013; Ikon et al., 2015; Li et al., 2015; Manganelli et al., 2015). Furthermore, CL modulates the activity of various carrier proteins involved in energy metabolism including the ADP/ATP and carnitine acyl-carnitine translocases (Kadenbach et al., 1982; Noel and Pande, 1986; Robinson, 1993; Jiang et al., 2000; Schlame et al., 2000; Koshkin and Greenberg, 2000, 2002; Vaz et al., 2003; Gu et al., 2004; Brandner et al., 2005). Finally, the polyunsaturated chains of CL would provide a shield against reactive oxygen species (ROS) that have the capacity to damage any type of biomolecules (McMillin and Dowhan, 2002; Kim et al., 2004; Heit et al., 2011; Gonzalvez et al., 2013; Chu et al., 2013, 2014; Hsu et al., 2015; Ikon et al., 2015; Li et al., 2015; Manganelli et al., 2015).

Pre-mature CL is synthesized at the matrix side of the IM as a saturated phospholipid from phosphatidic acid (PA) originating in the endoplasmic reticulum (ER) (Schlame and Haldar, 1993; Schlame et al., 2000). To carry out its different functions, CL needs to be unsaturated, which involves a deacylation-reacylation cycle, resulting in CL species containing mainly mono-unsaturated and di-unsaturated chains of 16-18 carbons (Schlame and Haldar, 1993; Hatch, 1998; Baile et al., 2014a). This remodeling activity is sustained by a cardiolipin-specific phospholipase (CLP1), which generates monolyso-CL (MLCL) and tafazzin (Bolhuis et al., 1991; Adès et al., 1993; Gedeon et al., 1995; Bione et al., 1996; Barth et al., 2004) (encoded by the nuclear gene *TAZ*), which re-acylates MLCL (Neuwald, 1997; Testet et al., 2005; Schlame, 2013).

Loss-of-function mutations in *TAZ* are responsible for Barth syndrome (BTHS), which is an X-linked recessive disorder characterized by cardiac and skeletal myopathies, growth retardation, hypocholesterolemia, 3-methyl glutaconic aciduria and increased susceptibility to bacterial infections due to cyclic neutropenia (Barth et al., 1983). BTHS is very often fatal in childhood as a result of cardiac failure or sepsis, and there is still no effective treatment (Barth et al., 1983; Bolhuis et al., 1991). Mitochondria from BTHS patients show multiple anomalies, including: (i) a reduced level of CL with a concurrent increase in monolysocardiolipin (MLCL) (Vreken et al., 2000; Schlame et al., 2003; Valianpour et al., 2005); (ii) abnormal ultrastructure; (iii) pleiotropic respiratory defects possibly due to impaired respirasome stability (Barth et al., 1996; McKenzie et al., 2006); (iv) increased production of ROS; (v) a reduced capacity to sustain apoptosis; and (vi) an abnormally high tendency to proliferate in cells, perhaps as a means to compensate for the compromised energy-transducing activity of taffazin-deficient mitochondria (Xu et al., 2005; Gonzalvez and Gottlieb, 2007; Dudek et al., 2013; Ferri et al., 2013; Gonzalvez et al., 2013).

Much of what we know about BTHS comes from studies in the yeast *Saccharomyces cerevisiae*, which is a convenient system for modelling mitochondrial disease mechanisms (Baile and Claypool, 2013; Lasserre et al., 2015). Studies in this yeast have helped to define how CL is synthesized and remodeled to maintain a homogenous and highly unsaturated acyl-chain composition, and how mitochondria are influenced by defects in these processes (Claypool, 2009; Joshi et al., 2009; Baile et al., 2014b; Mileykovskaya and Dowhan, 2014). Yeast strains lacking the homolog of the human *TAZ* gene (*taz1Δ*) showed substantial MLCL accumulation with a concurrent decrease in CL (Vaz et al., 2003; Gu et al., 2004; Testet et al., 2005; Claypool, 2009) and respired poorly when grown at elevated temperature (Schlame and Haldar, 1993; Schlame et al., 2000; Vaz et al., 2003; Gu et al., 2004; Brandner et al., 2005). These phenotypes were efficiently suppressed by expressing the human *TAZ* gene in *taz1Δ* yeast, which provided a simple assay to test the functional consequences of mutations found in BTHS patients. Most of these mutations proved, when expressed in yeast, to affect the association of tafazzin with the IM, making it susceptible to proteolytic degradation (Claypool et al., 2006, 2011).

The common respiratory growth defect observed in yeast models of mitochondrial disease provides a simple read-out to enable large-scale screens for genetic suppressors able to rescue mitochondrial dysfunction (Baile and Claypool, 2013; Lasserre et al., 2015). Even when mitochondrial dysfunction is severe enough to abolish respiratory growth, yeast offers the unique advantage that such mutants can be kept alive and propagated on fermentable substrates for the use in suppressor screens. A number of interesting findings have been reported using this approach. For example, it was found that disease-causing mt-tRNALeu(UUR) mutations are efficiently rescued in yeast by overexpressing factors involved in mitochondrial protein synthesis, including the translation factor EF-Tu (TUFM in humans) and various (cognate and non-cognate) aminoacyl tRNA synthetases (Montanari et al., 2008, 2010; Park et al., 2008; Rorbach et al., 2008; Sasarman et al., 2008). The suppressor activity of these factors was also observed in human cells carrying similar mutations and shown to be independent of their tRNA-charging function, indicating that the mutated mt-tRNAs recover their functionality because of chaperone-like RNA-protein interactions (Francisci et al., 2011).

Other interesting studies have revealed that yeast models of human diseases caused by defects in the assembly of ATP synthase (Schwimmer et al., 2005) or mutations in MPV17 (Dallabona et al., 2010), a protein of as-yet-poorly characterized function, are rescued by the overexpression of Odc1p, which is a mitochondrial carrier transporting Krebs cycle intermediates through the IM (Fiermonte et al., 2001). Since ATP synthase and MPV17 have different, apparently non-related, functions, and owing to the existence of phenotypic similarities in yeast conferred by defects in these systems and in *TAZ1* (see Discussion), we wondered whether Odc1p could also, when overexpressed, compensate for a lack in CL remodeling. The results reported here show that overexpression of Odc1p fully restores oxidative phosphorylation in *taz1Δ* yeast. This finding defines the transport of oxodicarboxylic acids across the IM as a potential therapeutic target for a large spectrum of mitochondrial diseases, including BTHS.

## RESULTS

### Construction, growth properties, phospholipid content and genetic stability of *taz1Δ* yeast

**Fig. 1.**
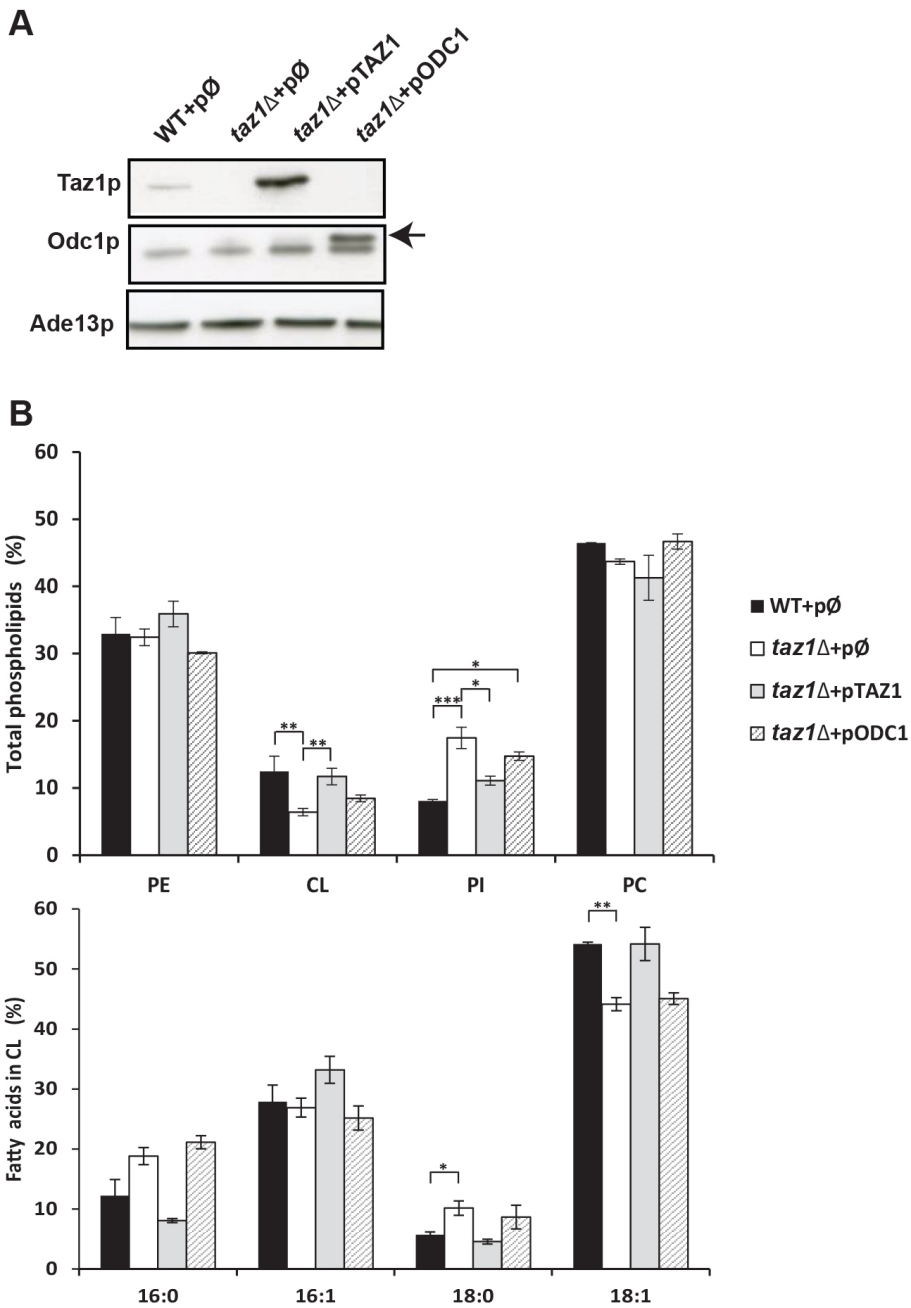
**Overexpressing Odc1p does not restore a normal phospholipid profile in *taz1Δ* yeast mitochondria.** (A) Steady-state levels of Odc1p and Taz1p. Total protein extracts were prepared from cells of the four analyzed strains (WT+pØ; *taz1Δ*+pØ; *taz1Δ*+p*TAZ1*; *taz1Δ*+p*ODC1*) grown at 36°C in a complete synthetic medium containing galactose and ethanol (see Fig. 2B for details). The proteins were separated by SDS-PAGE, transferred onto a nitrocellulose membrane and probed with antibodies against Odc1p and Taz1p, and the cytosolic Ade13p protein that was used as a loading control; 50 µg of proteins were loaded on each lane. The arrow indicates Odc1p. (B) Phospholipid composition and fatty acid chains in cardiolipin. Mitochondria were prepared from the analyzed strains grown as in A, and their lipids extracted and quantified. The top panel shows the relative contents of PE (phosphatidylethanolamine), CL (cardiolipin), PI (phosphatidylinositol) and PC (phosphatidylcholine) within each strain. The bottom panel gives the relative fatty acid chain composition of CL within each strain (16:0, palmitic acid; 16:1, palmitoleic acid; 18:0, stearic acid; 18:1: oleic acid). Statistical analysis was done with Kruskal-Wallis test (*P<0.05; **P<0.01; ***P<0.001). Data are expressed as mean±s.d. (*n*=4).

We first constructed a *taz1Δ* strain by replacing the *TAZ1* coding sequence with that of *TRP1*, which encodes a protein involved in tryptophan biosynthesis. The *taz1Δ* mutant was transformed with either the empty pRS426 plasmid (*taz1Δ*+pØ), which contains *URA3* as a yeast selection marker (Mumberg et al., 1994) or the same plasmid into which we had cloned the wild-type yeast *TAZ1* gene with its own promoter (*taz1Δ*+*pTAZ1*). As a control, we transformed the parental strain with pRS426 (WT+pØ). Western blot analyses of whole cell protein extracts confirmed the absence of Taz1p protein in *taz1Δ*+pØ (Fig. 1A). This protein was more abundant in *taz1Δ*+p*TAZ1* compared with WT+pØ, because pRS426 is a high copy number plasmid.

Consistent with previous studies (Testet et al., 2005), mitochondria isolated from our *taz1Δ* strain showed a significant decrease in CL level (about 50%) compared with the wild type, had a higher content in phosphatidylinositol (PI), while phosphatidylethanolamine (PE) and phosphatidylcholine (PC) accumulated normally (Fig. 1B, top). Also as expected, CL fatty acid chains were less unsaturated in *taz1Δ* versus WT mitochondria, with decreased levels in oleic (C18:1) and increased amounts of stearic (C18:0) acid chains (Fig. 1B, bottom). The *taz1Δ* mutant recovered a normal phospholipid profile upon transformation with the plasmid-borne *TAZ1* gene (*taz1Δ*+p*TAZ1*) (Fig. 1B).

**Fig. 2.**
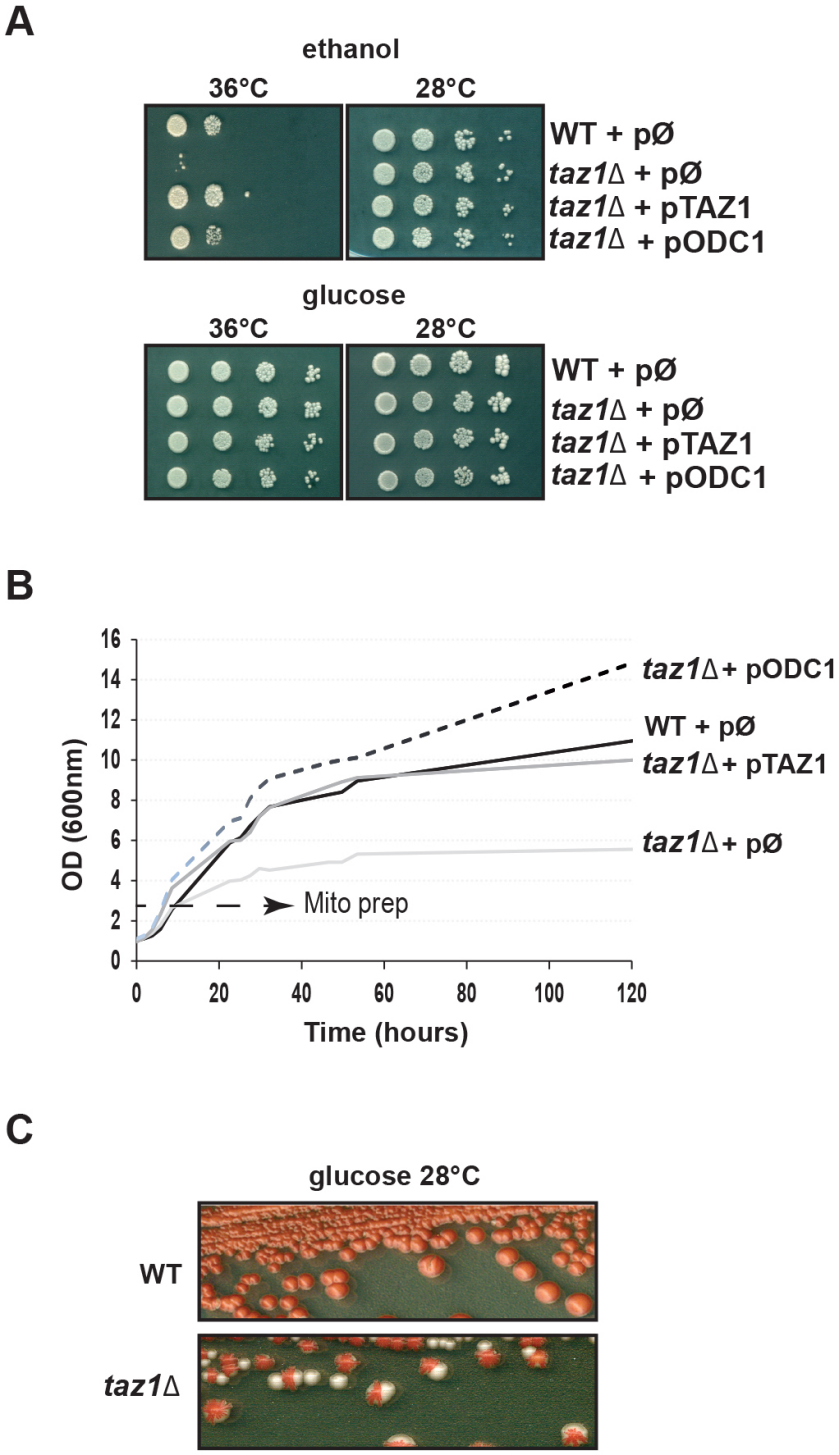
**Growth properties and genetic stability of yeast strains.** (A) Growth on glucose and ethanol. Cells from the four analyzed strains (WT+pØ; *taz1Δ*+pØ; *taz1Δ*+p*TAZ1*; *taz1Δ*+p*ODC1*) freshly grown at 28°C in complete synthetic medium (CSM) containing 2% glucose as a carbon source were serially diluted and spotted onto solid CSM+2% ethanol or CSM+2% glucose plates. The plates were photographed after 4 days of incubation at the indicated temperature. (B) Growth curves. Cells from the analyzed strains freshly grown in CSM+2% glucose at 28°C were inoculated into 50 ml of CSM+0.5% galactose+2% ethanol, and incubated at 36°C with shaking. Optical densities were measured over 1 week. The bioenergetics and biochemical investigations described in Figs 3-5 were performed with cells grown in these conditions (in 2 liters of medium) until 2-3 OD_600 nm_/ml as indicated by the dashed arrow. (C) Production of ρ^−^/ρ^0^ cells. Subclones of WT and *taz1Δ* mutant strains were grown on solid glucose plates with a limiting amount of adenine. Clones with a red color were picked and streaked on the same medium. The plates were photographed after 6 days of incubation at 28°C. As explained in the text, ρ^−^/ρ^0^ form entirely white colonies while those predominantly made of ρ^+^ cells are red.

Our *taz1Δ* strain (*taz1Δ*+pØ) grew poorly on respiratory carbon sources (ethanol) at elevated temperatures (36°C) whereas growth on non-fermentable substrates (glucose) looked normal (Fig. 2A). The respiratory growth deficiency was suppressed by the plasmid borne *TAZ1* gene (*taz1Δ*+p*TAZ1*) (Fig. 2A). We additionally tested growth of the strains in liquid complete synthetic medium (CSM) containing 0.5% galactose and 2% ethanol and devoid of uracil for plasmid maintenance (as used for the bioenergetics and biochemical investigations described below). Galactose is a fermentable substrate that does not elicit repression of mitochondrial function as glucose does. The four analyzed strains grew well in this medium at 28°C (not shown), whereas at 36°C, the growth of strain *taz1Δ*+pØ was much less efficient compared with the three other strains after consumption of the galactose present in the medium (Fig. 2B), which illustrates further the failure of the mutant to properly express mitochondrial function at 36°C.

Hundreds of nuclear genes required in yeast for mitochondrial function are important for mtDNA maintenance, either directly or indirectly (Contamine and Picard, 2000). Mutations in these genes result in the production of cytoplasmic *petite* cells issued from a large (>50%) deletion (ρ^−^) or totally devoid (ρ°) of mtDNA. To determine whether *TAZ1* is important for mtDNA stability, *taz1Δ* cells grown in glucose, conditions under which functional (ρ^+^) mtDNA is dispensable (except in rare *petite*-negative mutant contexts (Chen and Clark-Walker, 2000), were spread for single colonies on glucose plates with a limiting amount of adenine. The *ade2* mutated ρ^+^ cells form pink colonies owing to a red intermediate (AIR, aminoimidazoleribotide) of the adenine biosynthetic pathway, while *ade2* ρ^−^/ρ° cells form white colonies because this pigment can no longer be oxidized and remains white (Reaume and Tatum, 1949; Kim et al., 2002). *taz1Δ* yeast grown in glucose at 28°C had a relatively high tendency to produce ρ^−^/ρ° cells (50% vs <5% for the *WT*) (Fig. 2C). Since ρ^+^ *taz1Δ* yeast is respiratory competent at this temperature (Fig. 2A), it can be inferred that this mtDNA instability did not result from a failure in the expression of the energetic function of mitochondria (see Discussion). When grown in the synthetic galactose/ethanol medium at the non-permissive temperature (36°C), *taz1Δ* yeast produced much fewer (5-10%) *petites*, demonstrating that the mutant respiratory deficiency at elevated temperature did not result from a lack in functional mtDNA. The limited tendency of *taz1Δ* yeast to produce *petites* in these conditions is probably due to a strong counter-selection of these cells, whereas in pure fermenting conditions, they can grow efficiently.

### Bioenergetics in *taz1Δ* yeast

The impact of the loss of *TAZ1* on oxidative phosphorylation was investigated using mitochondria isolated from cells grown in synthetic galactose/ethanol medium lacking uracil at 36°C. The cells were harvested when the cultures reached a density of 2-3 OD_600nm_/ml after which the growth of the mutant (*taz1Δ*+pØ) became very slow compared with the wild-type (WT+pØ) and the mutant transformed with the plasmid borne *TAZ1* gene (*taz1Δ*+p*TAZ1*) (see Fig. 2B). As already mentioned, the mutant showed good mtDNA stability in these conditions, indicating that its respiratory growth defect at 36°C does not result from a lack of functional mtDNA.

#### Respiration

**Fig. 3.**
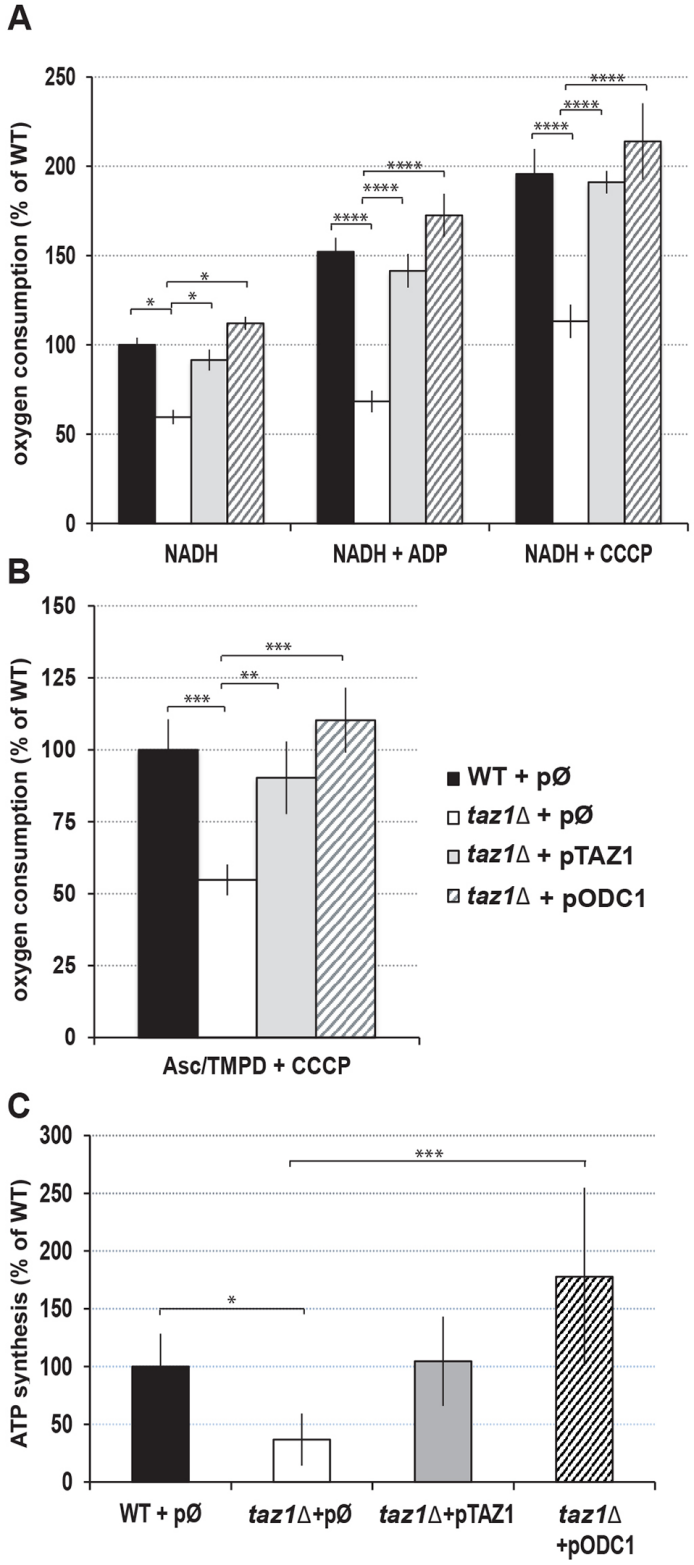
**Respiration and ATP synthesis in isolated mitochondria.** (A) Rate of oxygen consumption measured using NADH as an electron donor, alone (NADH, state 4), after further addition of ADP (NADH+ADP, state 3) or CCCP (NADH+CCCP, uncoupled respiration). (B) Rate of oxygen consumption measured using ascorbate/TMPD as an electron source in the presence of CCCP (Asc/TMPD+CCCP). (C) Rates of ATP synthesis measured using NADH as a respiratory substrate and in the presence of a large excess of external ADP. Mitochondria were prepared from cells grown as described in Fig. 2B. Statistical analysis was done with Tukey's test (*P<0.05; **P<0.01; ***P<0.001; ****P<0.0001). Data are mean±s.d. (*n*=4).

We first measured mitochondrial oxygen consumption using NADH as an electron donor, alone (basal, state 4 respiration), after further addition of ADP (state 3, phosphorylating conditions) or in the presence of the membrane proton ionophore CCCP (carbonyl cyanide mchlorophenylhydrazone; uncoupled respiration). Under state 4, the rate of respiration is controlled by the passive permeability to protons of the inner membrane (IM). Under state 3, it is generally accepted that most of the protons return to the matrix through the ATP synthase and that the contribution of the passive permeability to protons of the IM to respiration is then very small. In the presence of CCCP, the maintenance of an electrical potential (ΔΨ) across the IM is impossible and respiration becomes maximal. State 4 respiration was not increased in *taz1Δ* yeast compared with the two control strains (it was even a little decreased, see Fig. 3A), indicating that the absence of CL remodeling does not render the IM more leaky to protons. State 3 vs state 4 respiration was only slightly higher in the mutant (by about 10%), while mitochondria from the two *TAZ1*^+^ strains responded normally to ADP with a 50% increase in respiration rate (Fig. 3A). In the presence of CCCP, the mutant mitochondria respired more rapidly, but there was still an important (45%) deficit in oxygen consumption compared with the two CL-remodeling-competent strains (Fig. 3A). We also measured the activity of complex IV (CIV) (cytochrome *aa_3_*) using ascorbate/TMPD (N,N,N′,N′-tetramethyl-pphenylenediamine) as an electron donor system in the presence of CCCP. Here also, the mutant showed a substantially (50%) reduced electron transfer activity (Fig. 3B).

#### ATP synthesis

We next measured the rate of mitochondrial ATP synthesis using NADH as a respiratory substrate, in the presence of a large excess of external ADP. In these conditions, ATP is synthesized only by complex V (CV) using the proton-motive force generated by complexes III and IV (there is no complex I in *S. cerevisiae*). This activity was substantially reduced in the *taz1Δ* mutant by at least 50% compared with the controls, WT+pØ and *taz1Δ*+p*TAZ1* (Fig. 3C). Since state 3 respiration and ATP synthesis rates were decreased in similar proportions in the mutant, it can be inferred that the observed oxidative phosphorylation deficit mostly resulted from a slower ATP synthesis rate rather than a less-efficient coupling of the mitochondrial energy transducing system.

#### Membrane potential

**Fig. 4.**
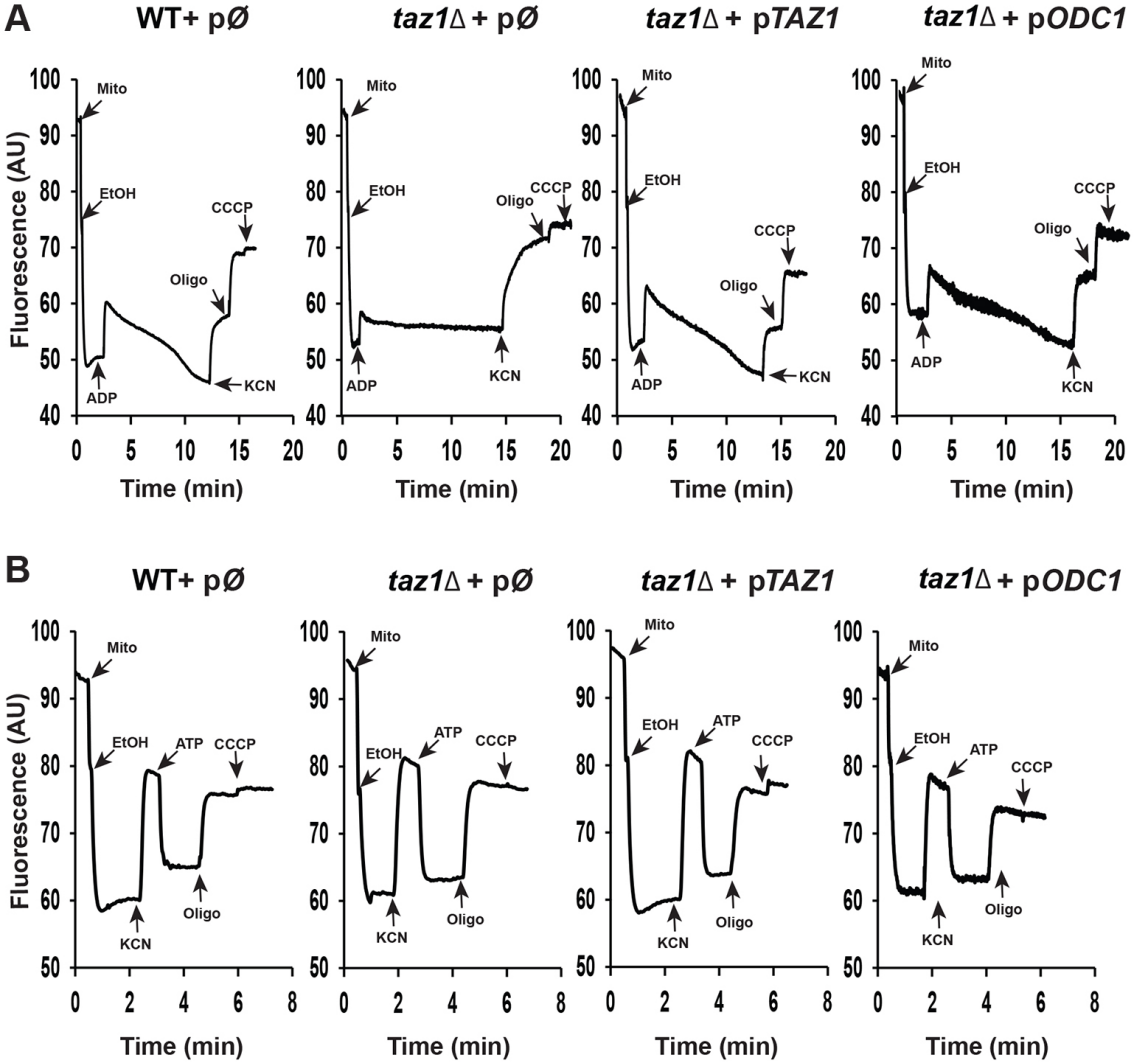
**Mitochondrial membrane potential.** Variations in mitochondrial ΔΨ were monitored by fluorescence quenching of Rhodamine 123. The effect of externally added ADP (A) and ATP (B) as well as 0.5 µg/ml Rhodamine 123, 75 µg/ml mitochondrial proteins (Mito), 10 µl ethanol (EtOH), 0.2 mM potassium cyanide (KCN), 50 µM ADP, 3 µM CCCP and 6 mg/ml oligomycin (oligo) on mitochondrial ΔΨ was followed. The fluorescence traces are representative of four experimental trials. The mitochondria used in these experiments were prepared from cells grown as described in Fig. 2B.

The consequences of a lack of CL remodeling on oxidative phosphorylation were analyzed further using Rhodamine 123. This is a fluorescent cationic dye that can be used to monitor changes in the IM electrical potential (ΔΨ) in intact mitochondria (Emaus et al., 1986). Increasing ΔΨ is followed by uptake of the dye inside the matrix space and concomitant fluorescence quenching. In a first set of experiments (Fig. 4A), we tested the capacity of externally added ADP to consume ethanol-induced ΔΨ. Despite their 50% reduced respiratory capacity (see above), ethanol induced a large ΔΨ variation in *taz1Δ* mitochondria, which is in agreement with previous studies showing that, in these conditions, the rate of respiration needs to be decreased by more than 80% to noticeably affect ΔΨ (Rak et al., 2007a; Kucharczyk et al., 2009). Normally, small amounts of ADP induce a transient fluorescence increase due to ΔΨ consumption by the ATP synthase during phosphorylation of the added ADP. This was indeed observed in mitochondria from the CL-remodeling-competent strains (WT+pØ and *taz1Δ*+p*TAZ1*), whereas *taz1Δ* mitochondria were mostly insensitive to ADP. KCN was then added to inhibit complex IV, which, in the control mitochondria, resulted in a rapid but partial ΔΨ collapse. The remaining potential was due to the pumping of protons by the F_O_ component of ATP synthase coupled to the hydrolysis by its F_1_ sector of the ATP that accumulated in the mitochondrial matrix during phosphorylation of the added ADP. Indeed, the remaining ΔΨ was lost upon addition of oligomycin. In *taz1Δ* mitochondria, the ethanol-induced ΔΨ was essentially collapsed in one rapid phase after addition of KCN, which further reflected their limited capacity to produce ATP (Fig. 4A).

In another set of experiments (Fig. 4B), we directly tested the functionality of ATP synthase using externally added ATP to ensure activity was independent of the respiratory chain (Fig. 4B). The mitochondria were first energized with ethanol to remove the natural inhibitory peptide (IF1) of the F_1_-ATPase. The mitochondrial membrane potential was then collapsed with KCN, and less than 1 min later (well before IF1 rebinding; Venard et al., 2003), ATP was added. Normally, the external ATP is counter-exchanged against ADP present in the matrix by the ADP/ATP translocase, which does not require any ΔΨ, and the ATP can then be hydrolyzed by F_1_ coupled to F_O_-mediated proton transport out of the matrix. In both *taz1Δ* and control mitochondria (WT+pØ and *taz1Δ*+p*TAZ1*), the addition of ATP promoted a large and stable fluorescence quenching of the dye that was reversed upon F_O_ inhibition with oligomycin. These results suggest that a lack in CL remodeling has limited, if any, impact on ATP synthase expression and functionality.

#### Assembly/stability of OXPHOS components

**Fig. 5.**
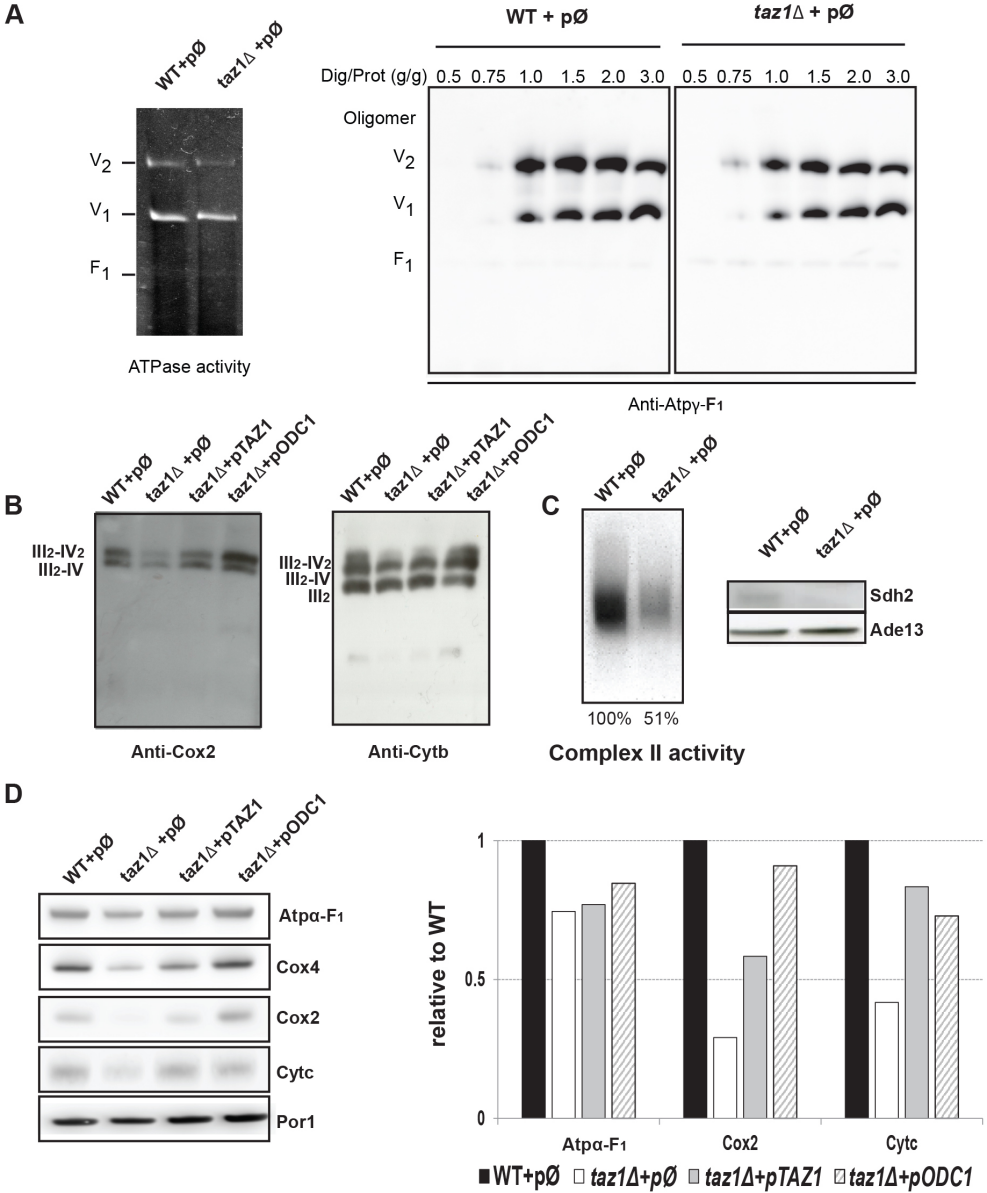
**BN-PAGE and SDS-PAGE analyses of mitochondrial proteins.** Experiments were performed with mitochondria isolated from strains WT+pØ, *taz1Δ*+ pØ, *taz1Δ*+p*TAZ1* and *taz1Δ*+p*ODC1* grown as described in Fig. 2B. (A) BN-PAGE analyses of ATP synthase. The left panel shows a BN-gel of mitochondrial proteins (50 µg) dissolved with 2 g of digitonin per g of proteins, where ATP synthase is revealed by its ATPase activity as dimers (V2), monomers (V1) or free F_1_ particles (F_1_). In the right panel, ATP synthase was analyzed in samples (50 µg) obtained after treating the mitochondria with increasing concentrations of digitonin, from 0.5 to 3.0 g per g of protein. After their electrophoretic separation and transfer onto a nitrocellulose membrane, the proteins were probed with antibodies against the γ-F_1_ subunit (ATP3) of ATP synthase. (B) BN-PAGE analysis of CIV and CIII. Mitochondrial proteins were extracted with 10 g digitonin per g of protein, separated by BN-PAGE (100 µg per lane), transferred onto a nitrocellulose membrane, and probed with antibodies against the COX2 subunit of CIV or the cytochrome *b* subunit of CIII. (C) BN-PAGE and SDS-PAGE analyses of CII. On the left panel, mitochondrial proteins were extracted with digitonin (10 g/g), separated by BN-PAGE, and assayed for in-gel complex II activity; in the right panel, 100 µg of total protein extracts were separated by SDS-PAGE, transferred onto a nitrocellulose membrane and probed antibodies against SDH2 and ADE13. (D) SDS-PAGE analyses. 100 µg of total mitochondrial proteins were separated by SDS-PAGE, transferred onto a nitrocellulose membrane and probed with antibodies against the indicated proteins. The right panel shows a quantification which as been done using ImageJ. Levels of COX2, ATPα-F_1_ and cytochrome *c* are related to the mitochondrial protein Por1p. The data are all relative to WT.

Blue native polyacrylamide gel electrophoresis (BN-PAGE) analyses of mitochondrial proteins extracted with various concentrations of digitonin revealed that full ATP synthase complexes similarly accumulated in *taz1Δ* and wild-type mitochondria, as monomeric and dimeric units (Fig. 5A). This further suggests that the activity of Taz1p is not crucial for ATP synthase expression and assembly, and association into dimers. By contrast, the respiratory system of *taz1Δ* yeast displayed substantial defects, with a rather severe lack in CII (Fig. 5C), CIII, CIV (Fig. 5B) and cytochrome *c* (Fig. 5D). In BN gels probed with COX2 and cytochrome *b* antibodies, most of the CIV present in *taz1Δ* mitochondrial protein samples was, as in the wild type, associated with CIII within CIII_2_-CIV_2_ and CIII_2_-CIV_1_ ‘supercomplexes’. The relatively weak response to cytochrome *b* antibodies of CIII_2_-CIV_2_ versus CIII_2_-CIV_1_ is certainly due to a much better accessibility of these antibodies to CIII when the CIII dimer is associated to only one CIV. For the same reason, ‘free’ CIII dimer responds even better to the cytochrome *b* antibodies. While this makes it difficult to quantify within a given mitochondrial sample the relative amounts of CIII- and CIV-containing protein complexes, comparison between samples makes sense, and it appears from Fig. 5B that CIII dimer was not more abundant in *taz1Δ* yeast compared with the wild-type strain, indicating that CL remodeling inactivation leads to similar decreases in the CIII and CIV content.

It can be inferred from these data that the slow-growth phenotype of the *taz1Δ* mutant on respiratory carbon sources at elevated temperature results from a lack in ATP synthesis owing to the defective expression of several components involved in the transfer of electrons to oxygen.

#### ROS production/accumulation

**Fig. 6.**
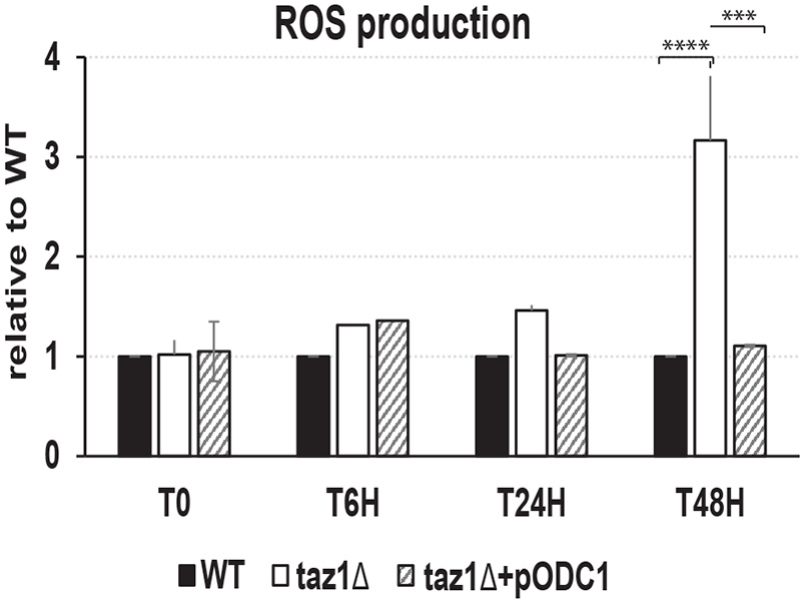
**ROS levels in yeast cells.** The four analyzed strains (WT+pØ; *taz1Δ*+pØ; *taz1Δ*+p*TAZ1*; *taz1Δ*+p*ODC1*) were grown as depicted in Fig. 2B. Cell samples were taken at the indicated times and analyzed by flow cytometry using dihydroethidium as a probe. The experiment was repeated three times for each strain. Statistical analysis was done with Tukey's test (***P<0.001; ****P<0.0001). Data are expressed as mean±s.d. (*n*=3).

Defects in the mitochondrial respiratory chain often result in a higher production/accumulation of reactive oxygen species (ROS) owing to the diversion of electrons from their normal pathway to oxygen. A lack in CL remodeling is known to provoke this phenomenon (Chen et al., 2008), which we also observed in our *taz1Δ* mutant (Fig. 6).

### Overexpressing *ODC1* fully restores oxidative phosphorylation in *taz1Δ* mutant

To test the capacity of Odc1p, when overexpressed, to compensate for a lack in CL remodeling, we transformed the *taz1Δ* mutant with a high copy number vector in which we have cloned *ODC1* with its own promoter (*taz1Δ*+p*ODC1*). As a result, Odc1p accumulated at more than 10-times higher levels compared with the untransformed mutant (Fig. 1A, arrow). Overexpression of Odc1p efficiently restored the ability of *taz1Δ* yeast to grow on respiratory substrates (Fig. 2A,B). The mitochondrial phospholipid profile of the modified yeast was mostly identical to that of *taz1Δ* cells that normally express *ODC1* (Fig. 1B). It can be inferred that the rescue was not induced by the restoration of CL remodeling.

Mitochondria isolated from *taz1Δ*+*pODC1* cells grown at elevated temperature respired (Fig. 3A,B) and produced ATP efficiently (Fig. 3C), displayed normal ΔΨ profiles (Fig. 4A), and had CIV, CIII and cytochrome *c* contents similar to those of wild-type mitochondria (Fig. 5B,D). Furthermore, the *taz1Δ* cells showed a normal production of ROS (Fig. 6). Thus, overexpression of Odc1p fully restored oxidative phosphorylation in CL-remodeling-deficient yeast cells.

### Oleic acid improves respiration dependent growth of *taz1Δ* yeast

**Fig. 7.**
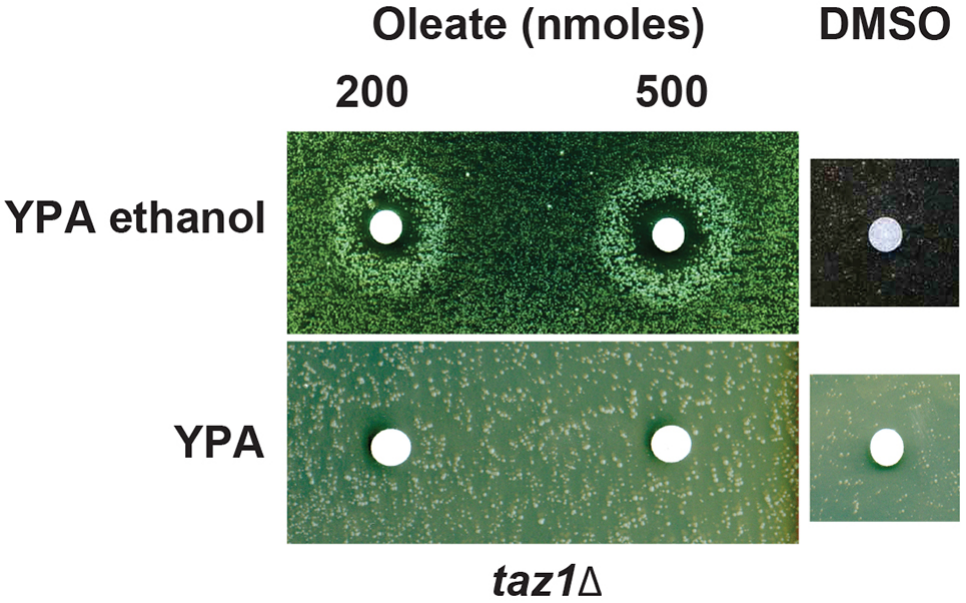
**Oleic acid improves respiratory growth of *taz1Δ* yeast.** Cells of *taz1Δ* yeast freshly grown in glucose were spread onto rich medium with or without ethanol as a carbon source. Small sterile filters were placed on the medium and oleate (dissolved in DMSO) or DMSO alone added to the filters at the indicated quantities. The plates were photographed after 5 days of incubation at 36°C.

Oleic acid (OA) is known to stimulate expression of *ODC1* (Tibbetts et al., 2002). Thus, if overexpressing *ODC1* can compensate for a lack in CL remodeling, we expected that this compound should also be able to do so. To test this hypothesis, *taz1Δ* cells were spread on solid ethanol medium (YPA ethanol) and exposed to filters spotted with different amounts of OA (Fig. 7). After several days of incubation at 36°C a halo of enhanced growth appeared around the filters. Oleate did not improve growth when the medium was devoid of ethanol (YPA), which demonstrates that the halos of enhanced growth resulted from a better utilization of respiratory substrates when *taz1Δ* yeast is exposed to the fatty acid.

## DISCUSSION

Consistent with previous studies using yeast as a model (Schlame and Haldar, 1993; Schlame et al., 2000; Vaz et al., 2003; Gu et al., 2004; Brandner et al., 2005), we found that a lack in CL remodeling due to inactivation of *TAZ1* compromises respiration in yeast cells exposed to elevated temperatures. Our biochemical and bioenergetics analyses revealed that a lack of several components (CII-IV and cytochrome *c*) involved in electron transfer to oxygen was possibly responsible for this instead of defects in the functioning of these systems. Thus, the CL levels still present (50% vs the WT) seem thus to be sufficient to support the function of the respiratory enzymes that accumulate in the *taz1Δ* mutant. At apparent odds with this, in a recent study (Baile et al., 2014b), while the absence of the *TAZ1* gene similarly affected oxygen consumption in yeast cells grown at 37°C, accumulation of respiratory chain complexes was not found to be diminished. Since this study and ours used the same host strain (W301-1A), background genetic differences are probably not responsible for this apparent discrepancy. A more likely explanation is that the two studies used very different growth conditions. In Baile et al. (2014b), the cells were grown in rich lactate medium whereas we used complete synthetic medium containing galactose and ethanol. Logically, the reduced content in electron transfer proteins we observed is probably not the primary event responsible for the impairment of respiration but rather a secondary, growth-condition-dependent consequence to some other functional impairment (see below).

To our knowledge, this is the first time that the influence of a lack of CL remodeling on the accumulation of cytochrome *c* has been tested. The reduced content of this cytochrome in the yeast tafazzin mutant is an interesting observation that further illustrates the importance of CL remodeling for preserving mitochondrial function. CL is known to be important for anchoring cytochrome *c* to the IM, which has been proposed to facilitate electron transfer from CIII to CIV (Orrenius and Zhivotovsky, 2005). It is possible that this interaction is impaired in *taz1Δ* yeast, and the released cytochrome *c* becomes more susceptible to proteolytic degradation. The high sensitivity of cytochrome *c* to oxidative damage possibly also contributes to the reduced accumulation of this protein in *taz1Δ* yeast owing to its enhanced propensity to produce ROS (Chen et al., 2008; this study). It will be of interest to determine whether a lack of cytochrome *c* contributes to the decreased rate of respiration in human CL-remodeling-deficient cells.

It has been established that CL is important for the association of respiratory complexes into supramolecular structures called respirasomes, and this organization is supposed to optimize respiration and minimize ROS production (Zhang et al., 2002; Pfeiffer et al., 2003; Claypool et al., 2008). There is evidence that respirasome destabilization occurs in BTHS patients, and this might contribute to the disease process (Barth et al., 1996; McKenzie et al., 2006; Gonzalvez et al., 2013). While a block in CL synthesis disrupts the interactions between CIII and CIV in yeast (Baile et al., 2014b), these complexes efficiently associate into supercomplexes in *taz1Δ* yeast (Baile et al., 2014b; this study). This indicates that the remaining CL levels (50% vs WT) in *taz1Δ* yeast are sufficient for making contacts between CIII and CIV, and that the compromised ability of the yeast tafazzin mutant to transfer electrons to oxygen does not result from a less-efficient association of these complexes. While the yeast and human mitochondrial energy-transducing systems are generally believed to be highly similar, there are some important differences such as the absence of CI in yeast, which could explain, at least in part, why the supra-molecular structure of this system is apparently more sensitive to a lack of CL remodeling in humans than in yeast. The compromised ability of BTHS patients to express CII possibly contributes to the disorganization of the electron transport chain owing to the well-known interactions between CI and CII (Wittig and Schägger, 2009).

While *taz1Δ* cells grew well in ethanol medium at 28°C (the optimal temperature for growing *S. cerevisiae*), they exhibited a high tendency to lose mtDNA when allowed to proliferate by fermentation (in glucose), i.e. under conditions where the presence of this DNA is not essential (Fig. 2C). This result indicates that the absence of CL remodeling does have an effect on yeast mitochondria at this temperature. A reasonable explanation is that the import into mitochondria of the systems involved in mtDNA maintenance becomes less efficient. In support of this hypothesis, it has been shown that biogenesis of the protein translocase (TOM) and sorting and assembly (SAM) machineries of the outer mitochondrial membrane is less efficient in CL-remodeling-deficient yeast cells grown at 28°C, and defects in these systems are synthetic lethal with mutations in the CL-remodeling pathway (Gebert et al., 2009; Sauerwald et al., 2015). The compromised ability of *taz1Δ* mitochondria to energize the IM (Fig. 4) could additionally impact protein import through the ΔΨ-dependent TIM23 machinery of the IM. Studies of other yeast mutants have shown that the systems involved in mtDNA maintenance are particularly sensitive to defects in mitochondrial protein import (Lefebvre-Legendre et al., 2003). The present study underscores the importance of CL maturation for efficient mitochondrial genome propagation.

Remarkably, oxidative phosphorylation and respiratory growth could be fully restored in *taz1Δ* yeast upon artificial overexpression of Odc1p, which is a protein that transports various Krebs cycle intermediates, preferentially α-ketoglutarate, across the inner mitochondrial membrane (Fiermonte et al., 2001). The level of Odc1p in *taz1Δ* yeast was not decreased compared with the wild-type strain (Fig. 1A), indicating that the respiratory deficiency of the mutant does not result from a failure to normally express/accumulate Odc1p. It is possible that the activity of Odc1p is affected by a lack in CL remodeling, in which case its overexpression would be beneficial not by stimulating but by preserving Odc1p function. While ODC1 expression can be modulated physiologically, with a 2- to 3-fold increase when yeast cells are switched from fermenting to respiratory conditions (Tibbetts et al., 2002), the levels of Odc1p in the mutant transformed with a plasmid-borne *ODC1* gene were at least 10-fold higher (Fig. 1A). Thus, yeast cells do not have the physiological capacity to increase the level of Odc1p in response to a lack of CL remodeling to a level sufficient to preserve oxidative phosphorylation. This can be achieved only artificially by way of genetic or pharmacological suppressors, which is an interesting finding that could help in developing therapeutic approaches against Barth syndrome.

Increasing the levels of Odc1p was previously shown to restore also respiration-dependent growth of a yeast mutant lacking a protein (Fmc1p) involved in the assembly of the F_1_ catalytic sector of CV at elevated temperatures (Lefebvre-Legendre et al., 2003; Schwimmer et al., 2005). Both complexes III and IV are strongly downregulated in this mutant. While CV assembly remained defective in *fmc1Δ* yeast when Odc1p was overexpressed, expression of CIII and CIV recovered much better (Lefebvre-Legendre et al., 2003; Schwimmer et al., 2005). As a result, the lack in CV was compensated for by an enhanced production of ATP by substrate-level phosphorylation directly coupled to the Krebs cycle (Schwimmer et al., 2005). As in the *fmc1Δ* mutant, increasing Odc1p accumulation restored a normal expression and activity of the electron transport chain in *taz1Δ* yeast. As a result, because of the high level of expression of CV in this mutant, ATP was again produced effectively through oxidative phosphorylation. Interestingly, Odc1p proved also, when overexpressed, to improve respiratory growth at elevated temperature of yeast cells lacking the gene (*SYM1*) encoding the homolog of human MVP17 (Dallabona et al., 2010). This is an IM protein of unknown function in which mutations have been associated with the hepatocerebral form of mitochondrial DNA depletion syndrome (Spinazzola et al., 2006). Whether the rescue of *sym1Δ* yeast on respiratory substrates correlates with improved expression/activity of the respiratory system is presently unknown.

How Odc1p, when overexpressed, compensates for defects in the oxidative phosphorylation system is certainly a complex process. Considering (i) the well-established function of this protein in the transport of α-ketoglutarate across the IM (Fiermonte et al., 2001), (ii) the kinetic control this transport exerts on oxidative phosphorylation (Rigoulet et al., 1985), and (iii) studies showing that some Krebs cycle enzymes like aconitase (Patil et al., 2013) and CII (Dudek et al., 2016; this study) are impaired in CL-remodeling-deficient cells, we hypothesize that the rescuing activity of Odc1p possibly results from the improved operation of the Krebs cycle. Some data reported in this study support the view that the respiratory deficiency of CL-remodeling-deficient cells does involve defects in this metabolic pathway: indeed, while the ATP synthesis rate was reduced by ∼50% in *taz1Δ* mitochondria using NADH as a respiratory substrate, these mitochondria almost completely failed to phosphorylate ADP when provided with ethanol (Fig. 4A). Obviously, an effective stimulator of the Krebs cycle requires a good capacity to transfer electrons to oxygen, and this capacity is substantially compromised in our *taz1Δ* yeast owing to a reduced presence of several components involved in this transfer (CIII, CIV and cytochrome *c*). Thus a two-pronged suppressor mechanism leading to an enhanced capacity to reduce NAD^+^ and a faster rate of NADH re-oxidation is required. In this respect, it should be noted that the functional state of mitochondria influences the expression of numerous nuclear genes through the so-called retrograde pathway as a means to provide the cell with a sufficient supply of metabolites entering the tricarboxylic acid cycle and anabolic reactions, and of proteins involved in oxidative phosphorylation (Jia et al., 1997; Liao and Butow, 1993; Butow and Avadhani, 2004). As we have shown, most of the genes encoding CIII and CIV subunits are strongly downregulated in *fmc1Δ* cells and regained a stronger transcriptional activity when exposed to chlorhexidine, a chemical that substantially improves the ability of these cells to grow on non-fermentable carbon sources (Couplan et al., 2011). It is possible that changing the flux of Krebs cycle intermediates across the IM in oxidation-impaired cells by overexpressing Odc1p secondarily activate the expression of genes encoding subunits of the OXPHOS system, as a means to balance the rates of reduction and re-oxidation of nicotinamide adenine nucleotides.

Our findings make the transport of oxodicarboxylic acids across the IM a potential target for the treatment of BTHS patients and other diseases caused by mitochondrial dysfunction. The rescue of *taz1Δ* (this study) and *fmc1Δ* (Couplan et al., 2011) yeasts by oleic acid – a molecule known to stimulate Odc1p expression (Tibbetts et al., 2002) – holds promise for the discovery of therapeutics targeting this key metabolite carrier.

## MATERIALS AND METHODS

### Growth media

The following media were used to grow yeast strains: YPAD, 1% (w/v) yeast extract, 2% (w/v) bacto peptone, 60 mg/l adenine and 2% (v/v) glucose; YPE, 1% (w/v) yeast extract, 2% (w/v) bacto peptone, and 2% (v/v) ethanol; CSMGE, 0.17% (w/v) yeast nitrogen base without amino acids and ammonium sulfate, 0.5% (w/v) ammonium sulfate, 0.5% (w/v) galactose, 2% ethanol and 0.8% (w/v) of a mixture of amino acids and bases from Formedium lacking uracil; WOABDF, 0.17% (w/v) yeast nitrogen base without amino acids and ammonium sulfate, 0.5% (w/v) ammonium sulfate, 0.5% (w/v), 2% (v/v) glucose, 50 mg/l adenine, 50 mg/l uracil, 50 mg/l histidine, and 50 mg/l leucine. Solid medium contained 2% (w/v) agar.

### Construction of *taz1Δ* yeast

*taz1Δ* yeast was constructed by replacing the open reading frame of *TAZ1* by that of *TRP1* in strain W303-1A (*MATa ade2-1 ura3-1 his311, 15 trp1-1 leu2-3,112 can1-100*), using a previously described procedure (Longtine et al., 1998). A *taz1::TRP1* DNA cassette was PCR amplified using a plasmid containing *TRP1* (pFA6a-TRP), and the primers Taz1-del-F (CAT TTT CAA AAA AAA AAA AAG TAA AGT TTT CCC TAT CAA cgg atc ccc ggg tta att aa) and Taz1-del-R (CCT CAT ACA TGC TAG TAT TTA CAC GAA TTT AAT TGC TTA AAT T gaa ttc gag ctc gtt taa ac). The nucleotides in capital letters correspond to TAZ1 flanking regions, those in lowercase are for TRP1 amplification. The PCR product was purified before transformation of W303-1A. Transformants were selected on WOABDF plates. The *taz1::TRP1* allele chromosomal integration was PCR-verified using a primer internal to *TRP1* (TRP1-466-R: CAG TCA GAA ATC GAG TTC CA) and two primers specific for *TAZ1* flanking regions (Taz1-Fbis: CGC CAG GAT CTG ACA GTA T; and Taz1-Rbis: TGA ATT CTA CCA GAT TGG TTA G).

### Plasmids

The *TAZ1* gene and its own promoter was PCR amplified from genomic DNA of W303-1A and primers *Sac*I-*Taz*1-F cccgagctccgCCA TTG TCT CTC CAA TTG GTG and *Xho*I-Taz1-R gcctcgagtcTCA ATC ATC CTT ACC CTT TGG. The *Sac*I and *Xho*I restriction sites included in the primers were used for cloning the PCR product into vector pRS426 (Mumberg et al., 1994). The plasmid containing *ODC1* (referred in this study as *pODC1*) was described in a previous study, in which it is referred to as pLL16 (Schwimmer et al., 2005).

### Bioenergetics experiments

Mitochondria were prepared by the enzymatic method as described (Guérin et al., 1979). Protein concentration was determined by the Lowry method (Lowry et al., 1951) in the presence of 5% SDS. Oxygen consumption rates were measured with a Clark electrode using 75 µg/ml of mitochondrial proteins in respiration buffer (0.65 M mannitol, 0.36 mM ethylene glycol tetra-acetic acid, 5 mM Tris-phosphate, 10 mM Tris-maleate, pH 6.8) as described previously (Rigoulet and Guerin, 1979) with 4 mM NADH, 12.5 mM ascorbate, 1.4 mM N,N,N′,N′-tetramethyl-pphenylenediamine (TMPD), 150 µM ADP and 4 µM carbonyl cyanide m-chlorophenylhydrazone (CCCP). Variations in transmembrane potential (ΔΨ) were evaluated in the same respiration buffer by monitoring the quenching of Rhodamine 123 fluorescence (0.5 µg/ml) using an FLX spectrofluorimeter (SAFAS, Monaco), as described previously (Emaus et al., 1986). ATP synthesis rate measurements were performed with 75 µg/ml mitochondrial proteins in respiration buffer supplemented with 4 mM NADH and 1 mM ADP in a thermostatically controlled chamber at 28°C (Rak et al., 2007b). Aliquots were withdrawn from the oxygraph cuvette every 15 s and the reaction was stopped with 3.5% (w/v) perchloric acid, 12.5 mM EDTA. The samples were then neutralized to pH 6.5 by adding 2 M KOH/0.3 M MOPS and ATP was quantified using a luciferin/luciferase assay (ATPLite kit from Perkin Elmer) and a LKB bioluminometer. The participation of the F_1_F_O_ ATP synthase in ATP production was assessed by adding oligomycin (2 µg/ml).

### BN-PAGE and SDS-PAGE

BN-PAGE was carried out as described previously (Schägger and von Jagow, 1991). Briefly, mitochondrial extracts solubilized with digitonin (the concentrations used are indicated in the legend of Fig. 5) were separated in a 3-12% acrylamide continuous gradient gel and F_1_F_O_ complexes were revealed in gel by their ATPase activity as described (Grandier-Vazeille and Guérin, 1996) or by western blot after transferred to poly(vinylidene difluoride) (Arselin et al., 1996). Complex II activity was revealed in a solution of 5 mM Tris-HCl, pH 7.4, 1 mg/ml NitroBlue Tetrazolium, 20 mM sodium succinate and 0.2 mM phenazine methosulfate. SDS-PAGE was performed as described (Rak et al., 2007b). The sources of antibodies and the dilutions at which they were used are: Atpγ-F_1_ (Jean Velours, IBGC, Bordeaux, France; 1:10,000), Atpα-F_1_ (Jean Velours; 1:10,000), cytochrome *c* (Stephen Manon, IBGC, Bordeaux, France; 1:10,000), Taz1 (Steven Claypool, Johns Hopkins University School of Medicine, Baltimore, USA; 1:5000), Ade13 (Benoít Pinson, IBGC, Bordeaux, France; 1:50,000), Cox2 (Abcam, ab110271; 1:500), Sdh2 (Cristina Dallabona, University of Parma, Italy; 1:5000), Cytb (Ulrich Brandt, Radboud University Medical Center, Nijmegen, The Netherlands; 1:5000), Cox4 (Abcam, ab110272; 1:1000) and Porin (Martin van der Laan, Institute of Biochemistry and Molecular Biology, Freiburg, Germany; 1:5000). Nitrocellulose membranes were incubated with 1:2500 diluted peroxidase-labeled antibodies (Promega). Immunological signal quantification was performed with ImageJ software (Gassmann et al., 2009).

### Phospholipid analysis

Mitochondrial lipids were extracted and analyzed as described (Testet et al., 2005). Briefly, mitochondria were treated with a mixture of chloroform/methanol (2:1, v/v). After centrifugation, the organic phase was separated and the remaining lipids were further extracted twice with chloroform. After the phase separation, the organic phases were pooled and evaporated to dryness. The lipids were then suspended in chloroform/methanol (2:1, v/v). Volumes equivalent to 50 µg of acyl chains were spotted on silica plates. Polar lipids were separated by one-dimensional TLC using chloroform/methanol/1-propanol/methylacetate/0.25% KCl (10:4:10:10:3.6 by volume) as a solvent (Vitello and Zanetta, 1978). The lipids were then located by immersing the plates in a solution of 0.001% (w/v) primuline in PBS, followed by visualization under UV. The silica gel zones corresponding to the various lipids (PE, CL, PI and PC) were then scraped from the plates and added to 1 ml of methanol/2.5% H_2_SO_4_ containing 5 µg of heptadecanoic acid methyl ester as a standard. After maintaining the lipids in the mixture at 80°C for 1 h, 1.5 ml of water and 400 µl of hexane were added. After centrifugation, hexane phase containing fatty acid methyl esters (FAMES) was isolated. Separation of FAMES was performed as described (Testet et al., 2005).

### ROS analyses

Cells at 0.4 OD units were taken from liquid cultures, pelleted in a microcentrifuge, suspended in 1 ml of phosphate-buffered saline (PBS) containing 50 µM dihydroethidium (DHE; Molecular Probes) and incubated at room temperature for 5 min. Flow cytometry was carried out on a Becton-Dickinson Accuri C6 model flow cytometer. The DHE fluorescence indicated was the direct output of the FL2A (red fluorescence) channel without compensation. A total of 100,000 cells were analyzed for each curve.

### Testing the influence of oleate on *taz1Δ* yeast respiratory growth

Exponentially growing cells at 0.125 OD units were spread homogeneously with sterile glass beads on a square Petri dish (12 cm×12 cm) containing solid YPE medium. Sterile filters were placed on the agar surface and spotted with oleic acid dissolved in dimethyl sulfoxide (DMSO) at a concentration of 100 mM. The plates were then incubated at 36°C for 6 days.
